# Plasmapheresis in post-COVID-19 myelitis: A case report

**DOI:** 10.5339/qmj.2024.19

**Published:** 2024-03-14

**Authors:** Witoon Mitarnun, Lisa Kongngern, Praewa Tantisungvarakoon, Theerapun Boonsayomphu, Nithit Tianchetsada, Tanluck Potchanapong

**Affiliations:** 1 Neurology Unit, Department of Internal Medicine, Buriram Hospital, Buriram, Thailand Email: miwitoon@gmail.com; 2 Department of Internal Medicine, Buriram Hospital, Buriram, Thailand

**Keywords:** severe acute respiratory syndrome coronavirus 2, COVID-19, myelitis, plasmapheresis, steroids

## Abstract

Background: Previous studies have delineated different neurological manifestations associated with coronavirus disease 2019 (COVID-19). Myelitis is identified as a rare neurological complication resulting from a COVID-19 infection. Limited information is available regarding the treatment of patients experiencing this condition.

Case report: This report extracts data from the medical record of a post-COVID-19 myelitis patient at Buriram Hospital and follows up prospectively on the patient’s symptoms after treatment.

A 61-year-old man, previously vaccinated for COVID-19 and with a history of hypertension and dyslipidemia, experienced progressive bilateral lower-extremity weakness (recorded as muscle strength grade 2/5 in both lower extremities) for 6 weeks. He had a mild case of COVID-19 2 months earlier, which resolved in 10 days without specific treatment. However, 2 weeks after being diagnosed with COVID-19, he developed weakness in his lower limbs, numbness below the nipple, and urinary retention. Spinal magnetic resonance imaging revealed multifocal longitudinal myelitis. Despite initial treatment with methylprednisolone, the patient showed no clinical improvement. Consequently, he underwent five cycles of plasmapheresis. Three months after discharge, a notable improvement was observed, with his muscle strength graded at 4/5 in both lower extremities and the resolution of sensory and urinary symptoms.

Conclusions: We presented the case of a COVID-19-vaccinated patient, in whom COVID-19 infection might have led to myelitis. We found promising results in treating prolonged COVID-19-related myelitis symptoms through the use of plasmapheresis.

## Introduction

Coronavirus disease 2019 (COVID-19) is an infectious respiratory illness caused by severe acute respiratory syndrome coronavirus 2 (SARS-CoV-2). First identified in December 2019 in Wuhan, China, it has since caused a global pandemic. COVID-19 impacts not only the respiratory system but also the gastrointestinal, cardiovascular, immunological, and neurological systems.^1–3^ Previous studies have described the various neurological manifestations of COVID-19, including stroke, cerebral venous sinus thrombosis, encephalitis, rhabdomyolysis, Guillain-Barré syndrome, optic neuritis, and myelitis.^3–8^

Myelitis is an immune-mediated central nervous system disorder characterized by inflammation of the spinal cord, leading to symptoms such as paralysis, sensory loss, and autonomic dysfunction. The incidence of myelitis ranges from 1.34 to 4.6 cases per million annually, with bimodal peaks in frequency—one in the age group of 10–19 years and another in the 30–39 years age range.^9^ Myelitis can result from various factors, including infection, systemic autoimmune or inflammatory diseases, paraneoplastic conditions, acquired central nervous system demyelinating diseases, and post-infectious or post-vaccination causes.^10^ In a study by Jeffery et al. involving 33 myelitis patients aged from 18 months to 82 years, 46% reported a preceding infection, with 73% being respiratory infections, 13% being gastrointestinal infections, and 13% displaying flu-like symptoms. Approximately 20–40% of infection-related cases are attributed to viral causes.^11^ Post-viral myelitis can be caused by different viruses, including herpesviruses, *Cytomegalovirus*, Epstein-Barr virus, *Flavivirus*, echovirus, hepatitis B virus, mumps virus, measles virus, and rubella virus.^12^ COVID-19-associated myelitis is a rare neurological complication with a global incidence of 0.5 cases per million among COVID-19 patients.^5^

The current recommendation for the initial treatment of myelitis advocates the use of high-dose steroids. Additionally, plasmapheresis is considered a second-line treatment, serving as rescue therapy for patients who do not respond to high-dose steroids.^12,13^ However, data on the standard treatment of post-COVID-19 myelitis are scarce, creating a knowledge gap. Therefore, in this report, the authors describe a case of post-COVID-19 myelitis that occurred during the later stages of the pandemic. Although the patient experienced prolonged symptoms, there was a positive response to plasmapheresis.

Approval was obtained from the Buriram Hospital Ethics Committee (IRB: BR0033.102.1/29) to publish this case report, and the patient provided informed consent.

## Case Description

A 61-year-old man with hypertension and dyslipidemia presented at Buriram Hospital, a government regional hospital located in Buriram province, Thailand, with progressive bilateral lower-extremity weakness over a 6-week period. Two months earlier (in December 2022, during the later stages of the COVID-19 pandemic in Thailand and worldwide), he had been diagnosed with mild COVID-19 after experiencing an acute febrile illness and cough. The diagnosis had been confirmed through a rapid antigen test for SARS-CoV-2 using a nasopharyngeal swab. He did not receive any antiviral or steroid treatments, and his COVID-19 symptoms had resolved within 10 days of home quarantine. Nevertheless, the patient complained of progressive bilateral lower limb weakness, numbness below the nipple, and urinary retention 2 weeks after the COVID-19 diagnosis. Previously, he received two doses of the ChAdOx1 nCoV-19 vaccine (Oxford–AstraZeneca COVID-19 vaccine), with the last dose having been administered in January 2022. The patient visited various clinics and hospitals, undergoing a two-day admission at the last one before arriving at our facility with an inconclusive diagnosis.

**Figure 1.**
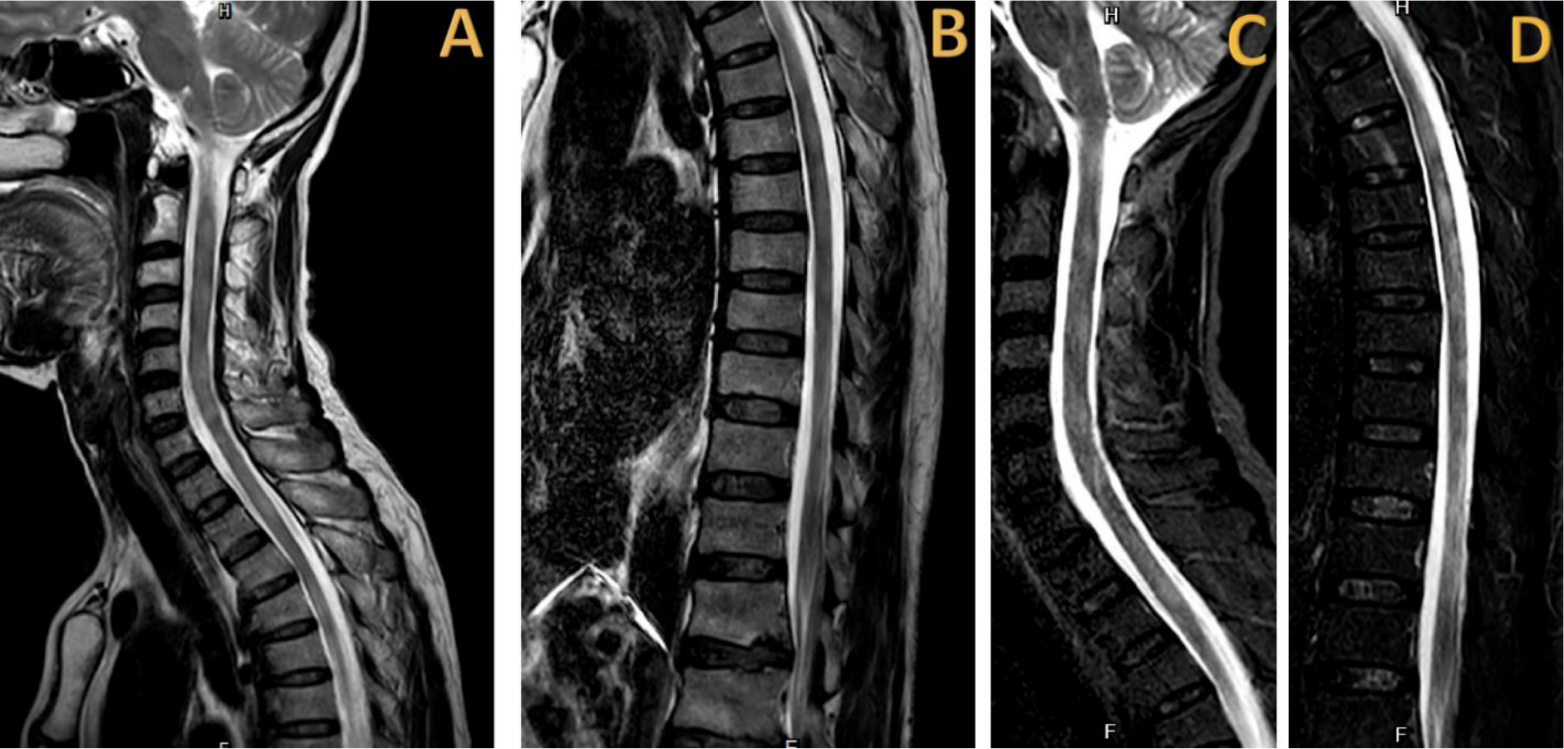
T2-weighted (A, B) and STIR-weighted (C, D) spinal magnetic resonance imaging illustrates multifocal regions of heightened signal intensity at the C2–C6, T2–T7, and T10–T11 levels, indicative of multifocal longitudinal myelitis. The findings are compatible with the patient’s symptoms.

**Figure 2.**
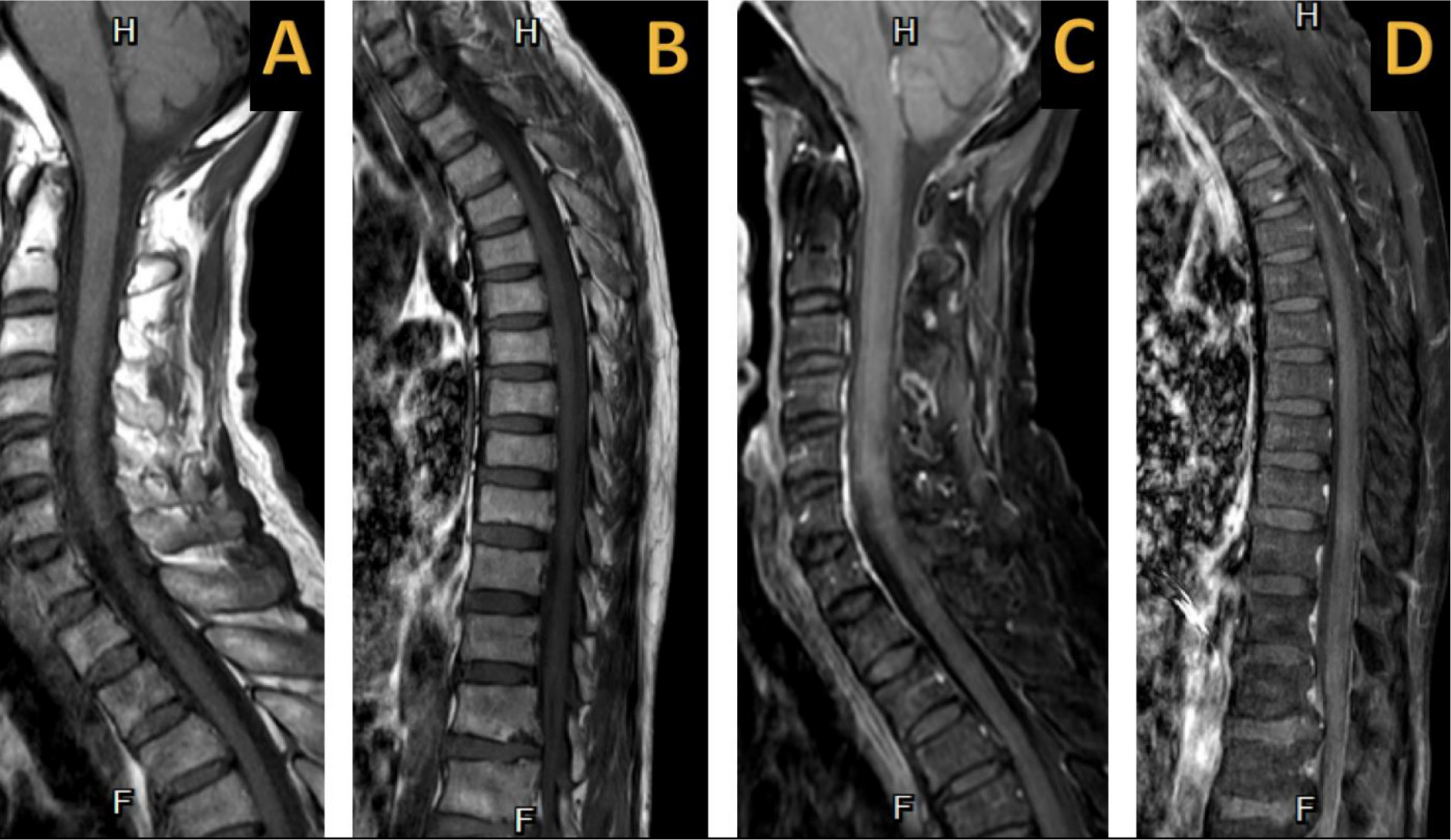
T1-weighted (A, B) and T1-weighted with gadolinium (C, D) spinal magnetic resonance imaging depicts the absence of abnormal signal intensity and gadolinium enhancement.

The patient was referred to the emergency department of our hospital and was subsequently admitted to the inpatient department of internal medicine. Upon examination at our hospital, the patient was afebrile with normal vital signs and already had a Foley catheter in place for urinary retention. Neurological examination revealed a reduced pinprick sensation below the T4 level, a muscle strength of 2/5 in both lower limbs, and no weakness or numbness in either upper extremity. All blood tests and nasopharyngeal swabs were conducted on the first day of admission. The nasopharyngeal rapid antigen test for SARS-CoV-2 and serum anti-SARS-CoV-2 IgM tests yielded negative results, while the immunochromatographic assay for anti-SARS-CoV-2 IgG showed positive results. The complete blood count, blood chemistry, renal function, and liver function test results were normal. Serological tests for hepatitis B, hepatitis C, and human immunodeficiency virus yielded negative results. The spinal magnetic resonance imaging (MRI), performed on the second day of admission, revealed multifocal regions of high signal intensity at the C2–C6, T2–T7, and T10–T11 levels on both the T2-weighted (Figure 1A and 1B) and STIR-weighted (Figure 1C and 1D) imaging sequences, without gadolinium enhancement on the T1-weighted images (Figure 2). Additionally, degenerative changes with disc bulging at L1–L2 and L2–L3 were noted, resulting in moderate central canal and bilateral foraminal stenosis. On the third day of admission, a lumbar puncture was performed, which revealed cerebrospinal fluid (CSF) protein and glucose levels of 32.8 mg/dL and 81.9 mg/dL, respectively, without any CSF pleocytosis (three white blood cells/mm^3^). Tests for serum aquaporin-4, serum myelin-oligodendrocyte glycoprotein antibodies, and CSF oligoclonal bands returned negative results. Consequently, the patient was diagnosed with probable COVID-19-associated myelitis. Initial treatment began on the third day of admission, starting with intravenous methylprednisolone (1 g/day) for 5 days, followed by oral prednisolone (50 mg/day). Despite the treatment, no clinical improvement was observed. Subsequently, the patient underwent five cycles of plasmapheresis, with the first cycle starting on the tenth day of admission. Plasmapheresis was done using fresh frozen plasma at 3 L per cycle. After a 3-week hospital stay, the patient was discharged with slight improvement, displaying a muscle strength of 3/5 in the right lower extremity, while the left lower extremity maintained the muscle strength of 2/5. Three months after discharge, the patient demonstrated improved muscle strength at 4/5 in both lower extremities, along with the resolution of sensory and urinary symptoms.

## Discussion

In the present case, despite having received two doses of the ChAdOx1 nCoV-19 vaccine (with the last one administered 11 months prior to the infection), which a previous study indicated had an overall estimated efficacy of 74.0%,^14^ the patient still contracted COVID-19. Two weeks after infection, he exhibited symptoms such as paraparesis, sensory impairment below T4, and urinary issues. The spinal MRI confirmed multifocal longitudinal myelitis, while tests for serum aquaporin-4, serum myelin-oligodendrocyte glycoprotein antibodies, serum hepatitis B antigen, and CSF oligoclonal bands yielded negative results. Overall, the findings were consistent with myelitis based on the diagnostic criteria for the condition.^10^ Additionally, the findings align with those of previous studies,^5,6^ further supporting the diagnosis of a probable case of COVID-19-associated myelitis.

The first case report of COVID-19-associated myelitis was described by Abdelhady et al.^4^ in 2020, followed by a case series of 20 patients conducted by Schulte et al.^5^ in 2021. The study reported that the average time between COVID-19 infection and myelitis onset was 10.3 days. The median age was 56 years, with a slight male predominance (60%). The CSF findings indicated an inflammatory response in 77.8% of the cases. Longitudinal myelitis typically emerged on the spinal MRI as cord lesions spanning an average of 9.8 vertebral segments in length. The findings noted in the present case are similar to those described previously.^5^ The pathogenesis of COVID-19-associated myelitis is still unclear. The authors hypothesize that the development of COVID-19-associated myelitis could occur through at least three mechanisms. Firstly, the virus directly invades and replicates within spinal cord neurons, facilitated by the presence of angiotensin-converting enzyme 2 (the primary entry receptor for SARS-CoV-2) on the membranes of the neurons.^15^ Secondly, indirect damage results from severe systemic illness or cytokine storm syndrome.^16,17^ Thirdly, a para- or post-infectious mechanism may be initiated by molecular mimicry and the cell-mediated immune (CMI) response.^17^ In the present case, COVID-19 symptoms were mild, and the onset of myelitis after infection occurred at 2 weeks, which is compatible with the onset of the CMI response. Therefore, the rational explanation for myelitis in our case could be a para- or post-infectious mechanism.

The current guideline^12,13^ recommends high-dose steroids for myelitis treatment, with plasmapheresis as a secondary intervention for non-responders. Data on treating COVID-19-associated myelitis are limited; however, findings from a systematic review show that 90% of patients with COVID-19-associated myelitis partially or completely recovered after immunotherapy.^5^ Previous studies^7,8,18–23^ have also suggested that patients with COVID-19-associated myelitis could benefit from plasmapheresis, with most experiencing at least partial recovery. Notably, Sarma et al.^22^ revealed that administering methylprednisolone followed by plasmapheresis resulted in nearly complete recovery. Consequently, high-dose steroids were administered to the patient followed by plasmapheresis. Three months later, the symptoms improved significantly. Although our findings are consistent with those of previous reports, notably, the patient experienced a longer duration of symptoms (6 weeks) in contrast to those previously documented.^7,8,18–23^ The incomplete recovery may be due to the long spinal cord lesions and long duration of myelitis symptoms before treatment initiation.

The present case report has some limitations. The presence of SARS-CoV-2 and anti-SARS-CoV-2 antibodies in the CSF was not tested owing to the unavailability of the tests, which prevented the exclusion of direct central nervous system invasion by SARS-CoV-2, and the case could not be diagnosed as a confirmed case of COVID-19-associated myelitis.^6^

## Conclusion

We presented a case of a 61-year-old man in whom COVID-19 infection might have led to myelitis. He experienced myelitis symptoms for 6 weeks. Despite initial methylprednisolone treatment, there was no improvement. However, after undergoing five cycles of plasmapheresis, significant improvement was observed three months later. Our findings not only support the concept of a post-viral immune response in COVID-19-associated myelitis and confirm the benefits of immunotherapy, including plasmapheresis, but also highlight the novelty that plasmapheresis remains beneficial even in cases of prolonged symptoms.

## Acknowledgments

We thank the patient for his cooperation.

## Author Contributions

Witoon Mitarnun developed the case study concept and design, edited the figure, and wrote and edited the article. Lisa Kongngern, Praewa Tantisungvarakoon, Theerapun Boonsayomphu, Nithit Tianchetsada, and Tanluck Potchanapong wrote and edited the article.

## Conflicting of Interests

The authors declared no potential conflicts of interest with respect to the research, authorship, and/or publication of this article.

## Ethical Approval

The present report was approved by the Ethics Committee of Buriram Hospital (IRB: BR0033.102.1/29).

## Informed Consent

The patient gave informed consent.
